# A novel *in vitro *bovine cartilage punch model for assessing the regeneration of focal cartilage defects with biocompatible bacterial nanocellulose

**DOI:** 10.1186/ar4231

**Published:** 2013-05-14

**Authors:** David Pretzel, Stefanie Linss, Hannes Ahrem, Michaela Endres, Christian Kaps, Dieter Klemm, Raimund W Kinne

**Affiliations:** 1 Experimental Rheumatology Unit, Department of Orthopedics, Jena University Hospital, Friedrich Schiller University Jena, Waldkrankenhaus "Rudolf-Elle" GmbH, Klosterlausnitzer Str. 81, D-07607 Eisenberg, Germany; 2 Jenpolymer Materials Ltd. & Co. KG, Technologie- und Innovationspark Jena - TIP, Wildenbruchstraße 15, D-07745 Jena, Germany; 3 TransTissueTechnologies GmbH, Charitéplatz 1/Virchowweg 11, D-10117 Berlin, Germany; 4 Institute of Macromolecular and Organic Chemistry, Friedrich Schiller University Jena, Lessingstr. 8, D-07743 Jena, Germany; 5 Transfer Group Polymet e.V., Wildenbruchstr. 15, D-07745 Jena, Germany; 6 Institute of Pharmacy, Department of Pharmaceutical Technology, Friedrich Schiller University Jena, Otto-Schott-Straße 41, D-07745 Jena, Germany

## Abstract

**Introduction:**

Current therapies for articular cartilage defects fail to achieve qualitatively sufficient tissue regeneration, possibly because of a mismatch between the speed of cartilage rebuilding and the resorption of degradable implant polymers. The present study focused on the self-healing capacity of resident cartilage cells in conjunction with cell-free and biocompatible (but non-resorbable) bacterial nanocellulose (BNC). This was tested in a novel *in vitro *bovine cartilage punch model.

**Methods:**

Standardized bovine cartilage discs with a central defect filled with BNC were cultured for up to eight weeks with/without stimulation with transforming growth factor-β1 (TGF-β1. Cartilage formation and integrity were analyzed by histology, immunohistochemistry and electron microscopy. Content, release and neosynthesis of the matrix molecules proteoglycan/aggrecan, collagen II and collagen I were also quantified. Finally, gene expression of these molecules was profiled in resident chondrocytes and chondrocytes migrated onto the cartilage surface or the implant material.

**Results:**

Non-stimulated and especially TGF-β1-stimulated cartilage discs displayed a preserved structural and functional integrity of the chondrocytes and surrounding matrix, remained vital in long-term culture (eight weeks) without signs of degeneration and showed substantial synthesis of cartilage-specific molecules at the protein and mRNA level. Whereas mobilization of chondrocytes from the matrix onto the surface of cartilage and implant was pivotal for successful seeding of cell-free BNC, chondrocytes did not immigrate into the central BNC area, possibly due to the relatively small diameter of its pores (2 to 5 μm). Chondrocytes on the BNC surface showed signs of successful redifferentiation over time, including increase of aggrecan/collagen type II mRNA, decrease of collagen type I mRNA and initial deposition of proteoglycan and collagen type II in long-term high-density pellet cultures. Although TGF-β1 stimulation showed protective effects on matrix integrity, effects on other parameters were limited.

**Conclusions:**

The present bovine cartilage punch model represents a robust, reproducible and highly suitable tool for the long-term culture of cartilage, maintaining matrix integrity and homoeostasis. As an alternative to animal studies, this model may closely reflect early stages of cartilage regeneration, allowing the evaluation of promising biomaterials with/without chondrogenic factors.

## Introduction

The unique anatomical structure of articular cartilage is characterized by avascularity, low cell density and very dense extracellular matrix [1,2]. Traumatic and osteoarthritis defects possess a very limited regeneration capacity, with dramatic loss of cartilage substance in the remaining tissue or complete loss of joint function.

Hence, the development of suitable treatments for articular cartilage defect regeneration is a major goal of modern orthopedic research. Several surgical procedures have been introduced to address this problem, for example, lavage, shaving, debridement, abrasion, microfracturing techniques [3], osteochondral autologous transplantation systems [3] and, as the present gold standard, the matrix-assisted, autologous chondrocyte transplantation (MACT) [4]. These techniques, however, mostly do not stop the progression of cartilage degeneration. One reason for the failure is that the regenerated tissue mainly consists of fibrous or osseus cartilage with functional and biomechanical properties clearly inferior to those of hyaline cartilage. This regenerated tissue shows early degradation and loss of function [5-7]. Concerning tissue or cell transplants, basic problems are the isolation of adequate quantities of biological material and the necessity to generate donor defects in healthy cartilage. A major problem is also the non-synchronized degradation of the resorbable cell-containing scaffold and regeneration of the damaged cartilage. Indeed, bioresorbable polymers are usually degraded in the body within a few weeks, whereas the reconstruction of fully functional cartilage usually requires months or even years.

Thus, alternative concepts and materials are clearly needed. One possibility is to employ the endogenous self-healing capacity of resident cartilage cells by using a cell-free and biocompatible, but non-resorbable cartilage implant, for example on the basis of bacterial nanocellulose (BNC). This material could serve as a mechanically stable, persistent scaffold for the migration of local cells into the defect-filling implant, which is then enriched by newly synthesized cartilage matrix.

BNC, synthesized by *Gluconacetobacter xylinum*, can be produced in many geometrical shapes and micro-structures [8,9] and is composed of nanoscale cellulose fibers (thickness 70 to 150 nm) with a tensile strength comparable to that of steel or Kevlar [10]. As a typical hydrogel, it has a water content of up to 99% and shows a moderate compression resistance and form stability. Importantly, the material causes no foreign body reactions [11] or cytotoxic effects [12] and is widely considered as highly biocompatible. The nanostructure of the BNC offers an attractive surface for the interaction with cells in terms of adhesion, proliferation and formation of new tissue [13].

BNC is employed for various medical applications [8,10,14-17] and may represent a promising orthopedic implant material for the regeneration of defects in tissues, such as meniscus [18], bone [19] or cartilage [20] [see Additional file 1]. In this context, BNC may help to circumvent the disadvantages of established therapies by being: 1) non-resorbable (stable scaffold during the whole regeneration time); 2) cell-free (one step surgery, no injury of healthy cartilage for a chondrocyte biopsy); 3) biocompatible; 4) producible in high quality and quantity; and 5) suitable for long-term storage.

In addition, chondrogenic key mediators can be combined with the biomaterial in order to support recruitment, proliferation, differentiation and matrix synthesis of chondrocytes by controlled release during the regeneration of cartilage defects. Besides growth factors, such as insulin-like growth factor-1 (IGF-1) [21-26] and fibroblast growth factor-2 (FGF-2) [23,27], transforming growth factor-β1 (TGF-β1) represents an especially attractive chondrogenic molecule. This is based on the induction of chondrogenic differentiation of mesenchymal stem cells [28-31], as well as its clear mitogenic [32-35] and matrix-inducing effects [33,34,36], although the latter point is still somewhat controversial [37-40]. Thus, TGF-β1 was chosen in the present study as a prototype molecule for the recruitment of resident cells, as well as for the induction of differentiation, proliferation and matrix synthesis.

The gold standard for the validation of new implant materials is the testing in established small or large animal models ([41]. Despite their unquestionable advantages, animal studies are time-consuming, expensive and may be ethically problematic. To reduce the need for animal experiments, we have established an *in vitro *model of cartilage regeneration with mature, adult bovine cartilage. This allows the long-term culture of cartilage tissue over several weeks under maintenance of matrix integrity and homoeostasis. In this model, early stages of cartilage formation can be simulated *in vitro *and used to analyze the suitability of biomaterials, such as non-resorbable, cell-free BNC, as cartilage implants in a standardized manner, including the evaluation of promising chondrogenic factors bound to the material. In comparison to previous models with cartilage or chondrocytes derived from immature calves [42,43] or pigs [44] known to have a much larger regeneration capacity [45], the present model works with material from adult bovine cartilage, which may more closely represent the situation in human osteoarthritis [2]. In addition, the present model employs the physiological surface of articular cartilage and is, in principle, suitable for high-throughput analyses in 48-/96-well plates.

## Methods

### Biosynthesis of bacterial cellulose (BNC)

In order to achieve cylindrical, rod shaped BNC hydrogels, vertical cultivation of *G. xylinus *(DSM 14666) was performed in glass tubes with an inner diameter of 3.6 mm. Several tubes were placed in a vertical orientation inside a beaker. A nutrient medium according to Hestrin and Schramm [46] was used for cultivation of the bacteria; the medium contained 20 g D-glucose, 5 g yeast extract, 5 g pepton, 3.4 g disodium hydrogen phosphate and 1.15 g citric acid per liter (HS medium). The HS medium was inoculated with a preculture of the bacteria in a volume ratio of 20:1 and cultivated within the glass tubes in the beaker. After culture for 14 days at 28°C, the BNC hydrogels were purified by treatment with 0.1 M sodium hydroxide solution for 30 minutes at 100°C, repeatedly rinsed with distilled water to pH 7 and finally autoclaved (121°C for 20 minutes using saturated steam and 2.1 bar pressure).

### Preparation of bovine cartilage, application of BNC inserts and embedding of constructs

Cartilage was obtained on the day of slaughter from six bovine knee joints (total of three mature, adult German Holstein Friesian Cattle, average age 24 months). Doughnut-shaped cartilage cylinders were aseptically dissected from the lateral facets of the trochlea/patella groove. To achieve this, first a biopsy punch with an inner diameter of 6 mm was used and, subsequently, a central defect within the 6 mm cartilage sample was created by applying another biopsy punch with an inner diameter of 2 mm. Finally, the cartilage was removed with a scalpel from the underlying bone (resulting height of the discs 1.3 ± 0.3 mm) and directly transferred into a dish containing culture medium (F12 Nutmix; ratio 1:1 (Invitrogen, Karlsruhe, Germany), with 100 μg/ml gentamycin, 5% FCS, and insulin-transferrin-selenium (ITS)-culture supplement (1:1000; final concentrations: 5 μg/ml insulin and transferrin, 5 ng/ml selenic acid; BD Biosciences, Heidelberg, Germany)). To remove contaminating blood, the cartilage discs were then washed once in PBS, also leading to a random distribution of cartilage discs derived from different locations in the bovine knee joint. A total of 96 cartilage samples were obtained from two femurs of one animal and randomly assigned to the two experimental groups (+/- TGF-β1).

Before application, each BNC cylinder was cut into five identical pieces using a scalpel and then applied press-fit with forceps into the defect of the cartilage discs.

**Figure 1:**
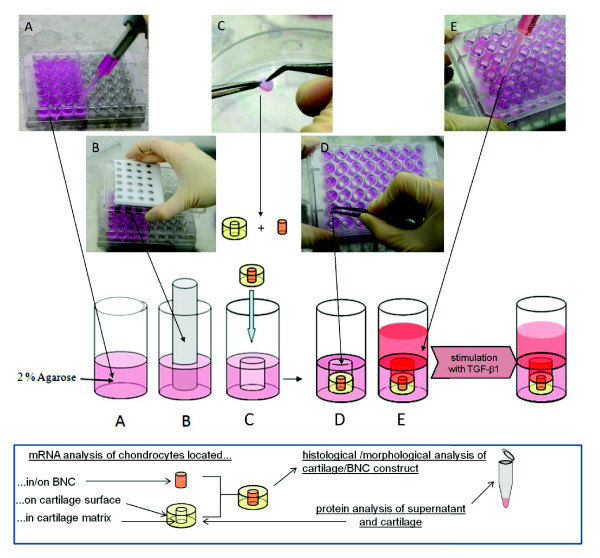
**Scheme of the *in vitro *model**. For embedding of the cartilage-BNC constructs, hot liquid agarose (2%) was added to the cavities of a 48-well plate **(A)**. Cylinders of defined size (5.8 mm) were created by inserting a metal-pin plate into the hot agarose and removing it after polymerization of the agarose **(B)**. The central defects of the cartilage discs were filled with the BNC material using forceps **(C) **and, after embedding the constructs into the agarose **(D)**, culture medium was added **(E)**. One part of the samples was stimulated with TGF-β1 at a concentration of 10 ng/ml. After *in vitro *culture, cartilage/BNC constructs were subjected to histological characterization. In addition, gene expression of chondrocytes isolated either from the BNC implant, the cartilage surface or the cartilage matrix was analyzed. At the protein level, the amount of cartilage components released into the supernatant as well as the remaining content in cartilage samples was quantified. BNC, bacterial nana-cellulose; TGF-β1, transforming growth factor-β1.

To ensure a reliable fixation, the cartilage/BNC constructs were embedded into the wells of a 48-well plate by adding a total of 300 μl hot liquid, 2% agarose (normal melting point; Invitrogen) into each well of a 48-well plate and subsequent generation of cylinders of a defined size (5.8 mm) by inserting a custom-made metal pin plate into the hot agarose (Figure 1). The cartilage discs were then fixed on the bottom of the preformed agarose cylinders; the use of agarose allowed sufficient diffusion of nutrients from the medium into the embedded cartilage matrix. The wells were filled with 500 μl culture medium and kept in an atmosphere of 37°C, 5% CO_2 _for two, four and eight weeks (Figure 1).

Three times a week, 550 μl of the culture supernatants were carefully replaced with fresh culture medium with/without TGF-β1. Supernatants were pooled over one week and stored at -20°C for further analyses. In each experimental group (+/- TGF-β1) 48 technical replicates from one animal were cultured in parallel for each time point (two, four and eight weeks), five were analyzed histologically, three were used for REM studies and, due to expected low amounts of RNA, the remaining 40 were pooled as four replicates of 10 samples each and processed for mRNA and protein analysis. This design was deliberately chosen in order to guarantee highly standardized conditions for the initial implementation of the model.

### Histology and immunohistochemistry

Five fresh, non-cultured cartilage discs, as well as cultured **c**artilage/BNC constructs, were directly fixed in PBS containing 4% paraformaldehyde and then subjected to paraffin embedding. For conventional histological staining and for immunohistochemical labeling, 4 μm thick tissue sections from the central part of the discs were mounted on superfrost plus slides (Menzel, Braunschweig, Germany). After deparaffinization in xylene for 30 minutes, sections were rehydrated through a gradient with decreasing proportions of ethanol. Cartilage morphology was analyzed after conventional hematoxylin/eosin staining (Hollborn, Leipzig, Germany). Proteoglycan content of the cartilage was assessed following Safranin-O staining and counterstaining with light green.

For immunohistological staining, tissue slices were subjected to different antigen retrieval treatments. For the detection of aggrecan, a demasking of the epitopes was performed by incubation with chondroitinase ABC (0.25 U/ml; Sigma-Aldrich, Taufkirchen, Germany) at 37°C for 90 minutes. For collagen type I and II staining, samples were treated with proteinase K (1:50 DAKO, Hamburg, Germany; code:S3004) for 15 minutes at room temperature. Endogenous peroxidase activity was blocked by 0.5% hydrogen peroxide in methanol for 15 minutes. The sections were then blocked for 30 minutes at room temperature with 10% serum/Tris-buffered saline (TBS). The respective sera were derived from the same species as the secondary antibody. Sections were incubated overnight at 4°C with unlabeled primary antibodies to bovine aggrecan (0.1 μg/ml, clone: MA85A95; GeneTex, Irvine, CA, USA), collagen type I (2 μg/ml, polyclonal rabbit sera; Acris, Herford, Germany) and collagen type II (10 μg/ml, clone II-4C11, Acris). Normal mouse or rabbit immunoglobulin G (IgG) was used in negative controls instead of the primary antibody. All antibodies were diluted in TBS containing 5% BSA. In the next step, binding was detected by incubating the sections for one hour with a secondary anti-mouse or anti-rabbit antibody coupled to horseradish peroxidase (HRP) or alkaline phosphatase (AP). The signal was visualized by incubation with hydrogen peroxide containing diaminobenzidine tetrahydrochloride chromogen (Sigma) for collagen type I and II and FastRed for aggrecan. The sections were washed with TBS between the different incubation stages and all steps were performed at room temperature unless otherwise stated. Sections were counterstained with hematoxylin, mounted with aquatex (Merck, Darmstadt, Germany) and examined by light microscopy.

### Scanning electron microscopy

In preparation for scanning electron microscopy (SEM) observation, three samples from each experimental group were fixed in a mixture of 2% (v/v) glutaraldehyde in 0.2 M sodium-cacodylate buffer (pH 7.2). After 72 hours, the samples were rinsed twice in 0.2 M sodium-cacodylate buffer and soaked in ethanol with ascending dilutions (50, 60, 70, 80, 90, 100% (v/v)) for water exchange. The ethanol was then replaced by acetone, the specimens dried in a critical point dryer (EMITECH K850; Emitech, Ashford, UK) and mounted with carbon tabs on aluminum stubs. They were then sputter-coated (EMITECH K500; Emitech) and analyzed using a SEM (XL-30 ESEM; Philips, Hamburg, Germany).

### RNA isolation

To obtain information on the matrix synthesis of chondrocytes from different sites of cartilage formation, RNA was isolated from: 1) cells migrated onto or into the BNC implant; 2) cells migrated onto the cartilage surface; and 3) cells located within the cartilage matrix (Figure 1). For the separate isolation of RNA from the three classified groups of cells, the BNC-cartilage constructs were removed from the wells and the BNC insert was carefully removed with forceps. A total of 40 inserts were collected, 10 inserts each pooled in four tubes containing 300 μl RLT-lysis-buffer (RNeasy^® ^Micro kit; Qiagen, Hilden, Germany), shortly vortexed, incubated for 15 minutes and stored at -80°C for subsequent RNA isolation.

The empty cartilage cylinders were treated for one minute in a tube with 600 μl lysis buffer under continous shaking to obtain the RNA from cells migrated onto the cartilage surface. After removal from the tube, cartilage discs were washed twice with PBS to remove remaining lysis buffer. Lysed cell fractions and cartilage discs were stored at -80°C until further use.

Before RNA isolation from cartilage, the shock-frozen cartilage (10 discs for each experimental group) was pulverized in a microdismembrator (Braun, Melsungen, Germany), as described previously [47]. Subsequently, RNA was extracted by resuspension of the powder in 600 μl RLT lysis buffer containing carrier RNA and centrifugation at 8,000 rpm at room temperature for two minutes. Total RNA of the cartilage discs and the lysed cell fractions (cells on cartilage surface and on BNC insert) was then isolated using the RNeasy^® ^Micro kit according to the supplier's instructions (Qiagen; including a DNase digestion).

### Reverse transcription and qPCR

Total RNA eluate (12 μl) was primed with Oligo(d)T and reverse-transcribed for one hour at 42°C using SuperScript-II reverse transcriptase (Invitrogen).

qPCR reactions were done as previously described [47] with PCR products as standards for the quantitation of bovine AGGRECAN, COLLAGEN TYPE I and TYPE II and the housekeeping gene ALDOLASE. qPCR was performed on a mastercycler 'realplex2' (Eppendorf, Hamburg, Germany) with HotMaster Taq (Eppendorf) and the primer pairs and PCR conditions presented in Table 1. The relative concentrations of cDNA present in each sample were calculated by the software using the standard curves. In order to normalize the amount of cDNA in each sample and to guarantee the comparability of the calculated mRNA expression in all analyzed samples, the housekeeping gene ALDOLASE was amplified and the relative cDNA amount normalized on the basis of these results. Product specificity was confirmed by melting curve analysis and initial cycle sequencing of the PCR products.

**Table 1:** Primers, product length and specific amplification conditions for qPCR.

Gene	Primer upstream(5'→3')	Primer downstream(3'→5')	Accession number	Product length in bp	T annealing	Melting T product
Aldolase	5´-TCATCCTCTTCCATGAGACACTCTA-3´	3´-ATTCTGCTGGCAGATACTGGCATAA-5´	NM_000034	314	58°C	88°C

Aggrecan	5´-CAGAGTTCAGTGGGACAGCA-3´	3´-AGACACCCAGCTCTCCTGAA-5´	NM_173981	189	60°C	84°C

Coll II	5´-CATCTGGTTTGGAGAAACCATC-3´	3´-GCCCAGTTCAGGTCTCTTAG-5´	NM_001001135	600	61°C	83°C

Coll I	5´-AGCCAGCAGATCGAGAACAT-3´	3´-ACACAGGTCTCACCGGTTTC-5´	NM_001034039	185	60°C	86°C

General amplification protocol (40 cycles): initial denaturation for two minutes at 95°C; denaturation for 15 seconds at 95°C, specific primer annealing temperature (see above) for 15 seconds, amplification at 68°C for 20 seconds, additional heating step to 5°C below the melting temperature of the PCR product (see above). General melting curve protocol (one cycle): denaturation for one second at 95°C; cooling to 5°C above the primer annealing temperature (holding for 10 seconds); heating to 95°C (0.1°C/second); final cooling for five minutes at 40°C. Col = collagen. qPCR = quantitative polymerase chain reaction.

### Extraction of proteins from cartilage

Cartilage proteins were extracted from the eluated lysates following RNA isolation using acetone precipitation according to the supplier's instructions of the RNeasy^® ^Micro kit (Qiagen). Briefly, one volume of sample was suspended in four volumes of ice-cold acetone, incubated for one hour at -20°C, and, after centrifugation at 8,000 × g and 4°C for 10 minutes and decanting of the supernatant, the precipitate was dried and stored at -20°C. Prior to protein analysis, samples were resuspended in 1 ml of 50 mM Tris-buffer (pH 7.6). Subsequently, the proteins in the cartilage powder remaining after RNA isolation, were solubilized for 48 hours at 4°C under continous shaking by an incubation with 10 volumes of 4 M GuHCl in 0.05 M sodium actetate (pH 6.0) including 1 mM ethylenediaminetetraacetic acid (EDTA), 10 μg/ml pepstatin A and 1 nM iodoacetamide. After centrifugation at 12,000 × g and 4°C for 30 minutes, the protein-containing supernatant was applied to ultrafiltration-tubes (MWCO 3000; Millipore/Amicon, Billerica, MA, USA), centrifuged at 4,000 rpm for two hours at 4°C, washed with 50 mM Tris-buffer (pH 7.6) containing proteinase inhibitors (1 mM EDTA, 10 μg/ml pepstatin A and 1 nM iodoacetamide) and finally subjected to protein elution in 500 μl of the 50 mM Tris-buffer.

For the assay-based analysis, both the precipitated proteins from the lysate and the extracted proteins from the cartilage powder were analyzed and the total content of the specific protein in the cartilage samples expressed as the sum of the lysate and the extracted protein.

The mean wet weight (*ww*) of the cartilage samples, as assessed in initial analyses, was 0.1373 ± 0.02 g per cartilage disc (*n *= 25) and was used as the basis for the expression of the results as 'quantity of the specific protein/g cartilage'.

### Quantification of glycosaminoglycans

The amount of sulphated glycosaminoglycans released from cartilage into the supernatant during culture, as well as the remaining content in the cartilage following culture, was quantified using the dimethylene blue-binding (DMB) assay, first described by Chandrasekhar [48]. Briefly, 50 μl of pooled supernatant and extracted/precipitated proteins, respectively, were applied to microtiter plates with or without dilution in 0.05 M sodium acetate-buffer (pH 6.8). After addition of 15 μl 2.8 M GuHCl solution and 200 μl DMB reagent (containing 1.9-dimethylene blue (16 μg/ml), 0.03 M sodium formiate, 0.2% formic acid; pH 6.8), absorption was read at 525 nm. A dilution series of a bovine nasal septum extract (Sigma) was used for the generation of a standard curve and calculation of the results.

### ELISA

The supernatants of cartilage-BNC cultures and precipitated/extracted cartilage proteins were screened for the amount of newly synthesized collagen (CPII; Ibex, Montreal, Canada), aggrecan (CS846; Ibex), collagen type II (MD Bioproducts, Egg, Switzerland) and cleaved collagen (C12C; Ibex). The commercially available ELISAs were performed according to the manufacturers' instructions.

### Micromass cultures of cells isolated from BNC, cartilage surface and cartilage matrix

In separate experiments, cartilage/BNC constructs were cultured for eight weeks with or without the addition of TGF-β1. Subsequently, the BNC inserts were removed from the cartilage cylinders and both were placed in separate dishes containing culture medium. In parallel, some cartilage cylinders without BNC inserts were subjected to cell isolation by enzymatic digestion of the cartilage. For this purpose, cartilage was incubated for one hour at 37°C and 5% CO_2 _in serum-free (D)MEM/F12 Nutmix ((D)MEM/F12; Invitrogen) containing 0.1% pronase E (Sigma-Aldrich, Taufkirchen, Germany) in a spinner flask for fine mincing and digestion. After two further washes, overnight enzymatic digestion was performed at 37°C in 0.05% collagenase P (Roche Diagnostics, Mannheim, Germany) in (D)MEM/F12 media supplemented with 5% FCS. Cells were separated by filtration through a 50 mesh sieve, washed twice in (D)MEM/F12 containing 5% FCS and antibiotics, and then, cells were seeded in culture dishes. Media were exchanged three times a week.

After reaching the required amount of cells, high-density cultures of chondrocytes isolated by 'outgrowth-cultures' from the BNC and cartilage surface and after enzymatic digestion of cartilage were generated by centrifugation to form a pelleted high-density culture. Stabilization of the chondrogenic phenotype/chondrogenic differentiation was induced for two weeks with (D)MEM-medium supplemented with ITS (Sigma-Aldrich, Taufkirchen, Germany) and 10 ng/ml TGF-β1 (R&D Systems, Wiesbaden, Germany). In non-induced controls, a basal medium without TGF-β1 supplementation was used. The medium was exchanged every other day. For histological and immunohistochemical analyses, high-density cultures were embedded in optimum cutting temperature (OCT) compound, frozen, and cryosections (thickness 6 μm) were prepared. Proteoglycans were visualized by staining with Alcian Blue 8GS (Roth, Karlsruhe, Germany) at pH 2.5. For immunohistochemical analysis of type II and type I collagens, cryosections (6 μm) were incubated for one hour with primary antibodies (rabbit anti bovine type II collagen, or rabbit anti bovine type I collagen; both Acris). In parallel, sections were incubated for one hour with rabbit IgG (DAKO, Hamburg, Germany) as controls. Subsequently, sections were processed using the EnVision System Peroxidase Kit (DAKO) according to the manufacturer's instructions, followed by counterstaining with hematoxylin (Merck, Darmstadt, Germany). Sections incubated with rabbit IgG showed no color reaction and documented the specificity of the type II and type I collagen antibodies and the peroxidase detection system.

## Results

### Morphology of cultivated cartilage BNC constructs

**Figure 2:**
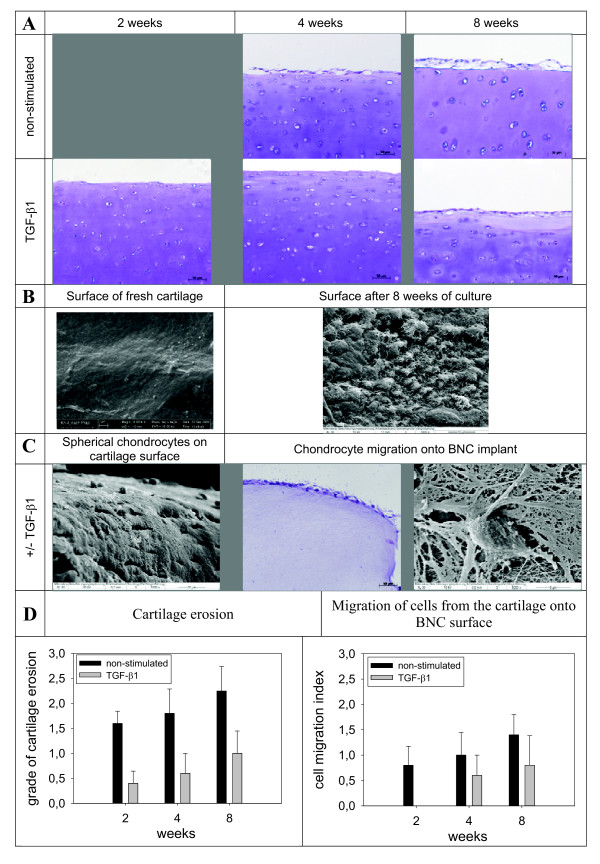
**Cartilage integrity and migration of chondrocytes out of the cartilage matrix**. (A) Histological analysis of cartilage surfaces of non-stimulated and TGF-β1-stimulated cartilage/BNC constructs after *in vitro *culture for two, four and eight weeks. The superficial layer of non-stimulated samples shows clear signs of beginning erosion and mobilization of chondrocytes. In contrast, the surface of TGF-β1-stimulated samples appears rather smooth and intact. Magnification: 200 x. **(B) **Scanning electron microscopy images display the smooth surface of fresh cartilage in contrast to uneven cartilage surface after eight weeks of culture, most probably caused by the removal of the lamina splendens. Magnification 2,000 x. **(C) **Migration of chondrocytes out of the dense cartilage matrix onto the cartilage surface and the BNC. Magnification left panel: 1,000 x, center panel: 200 x, right panel: 5,000 x. **(D) **Semiquantitative analysis of cartilage erosion and migration of cells from cartilage on the BNC. Stained histological sections were evaluated and scored with 0 to 3 points. Degree of erosion 0 = smooth cartilage surface, 1 = loss of lamina splendens, 2 = moderate erosion of superficial cartilage, 3 = massive erosion with complete loss of cartilage surface; degree of cell migration onto the BNC: 0 = BNC without cells, 1 = single adherent cells (<5/cartilage section), 2 = several adherent cells (<20/cartilage section), 3 = confluent cell layer on BNC. Values are shown as mean ± SEM for *n *= 5 technical replicates each. BNC, bacterial nana-cellulose; SEM, standard error of the mean; TGF-β1, transforming growth factor-β1.

Due to its enormous swelling capacity, a tight lateral bonding of the BNC insert to the cylindrical defect was achieved [see Additional file 2]. Despite the relatively long culture period of up to eight weeks, resident cartilage cells showed vital morphology without signs of alterations and positive nuclear staining, thus pointing to suitable culture conditions (Figure 2A). Interestingly, cartilage zones located close to the edge of the defect were characterized by the appearance of proliferation-induced cell clusters as a possible reaction to the initial mechanical tissue disruption (data not shown). The matrix integrity of the cartilage seemed to be largely unaffected during the whole culture period, except for a detachment of the superficial layer, presumably the lamina splendens, from the underlying tissue and a subsequent demasking of cartilage matrix structures (Figure 2B). TGF-β1 seemed to slow down the process of superficial delamination throughout the entire culture period of eight weeks. Increased delamination in non-stimulated samples was accompanied by augmented migration of cells onto the surface of the cartilage and the BNC implant (Figure 2 A, C), suggesting that the matrix erosion leads to a loosened network around the chondrocytes and to active emigration of the cells. Cells attached to the BNC implant showed a rather fibroblastic phenotype with flattened cell bodies and long cytoplasmatic protrusions (Figure 2C). Notably, there was no immigration of chondrocytes into the central area of the BNC, possibly due its relatively small pores (diameter 2 to 5 μm; Figure 2C). Semiquantitative analysis revealed that cartilage erosion and cell migration was clearly increased in non-stimulated versus TGF-β1-stimulated samples and became more pronounced with longer culture periods (Figure 2D).

### Matrix metabolism in cultivated cartilage BNC constructs

#### Localisation, content and release of proteoglycans

**Figure 3:**
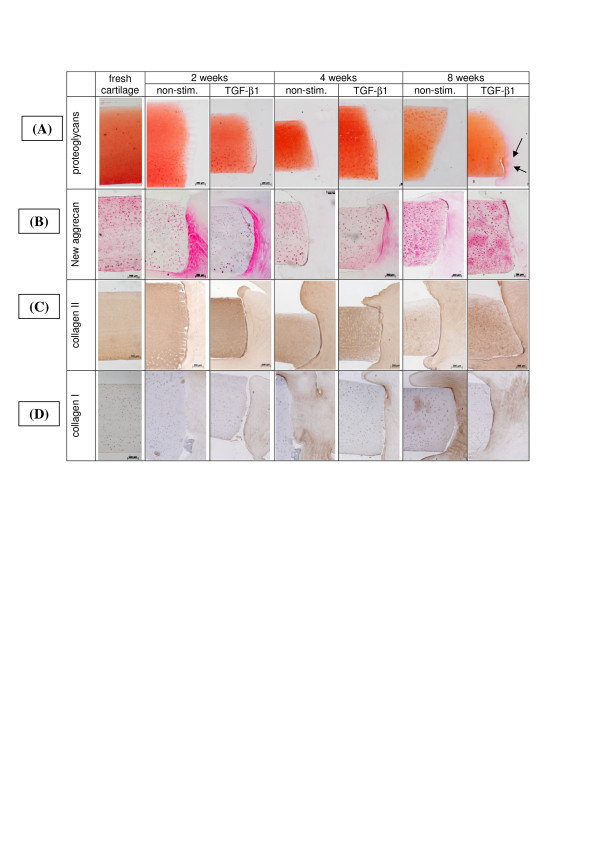
**Histological sections following conventional and immunohistological staining for the detection of cartilage matrix molecules**. Proteoglycan content was assessed in fresh and cultured (+/- TGF-β1) cartilage, as well as in the BNC by safranin-O staining **(A)**; aggrecan neosynthesis was shown using an antibody directed against a neoepitope on aggrecan molecules **(B)**. In addition, specific staining for collagen type II **(C) **and type I **(D) **was performed. Magnification 40 x. BNC, bacterial nana-cellulose; TGF-β1, transforming growth factor-β1.

**Figure 4:**
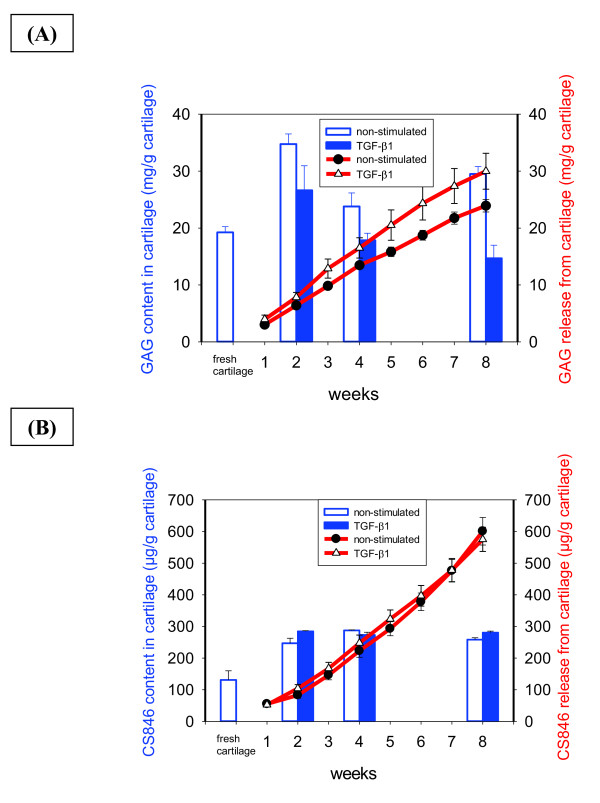
**Quantitative analysis of proteoglycans and the aggrecan neoepitope CS846**. **(A) **Proteoglycans were quantitatively assessed in fresh and cultured (+/-TGF-β1) cartilage, as well as in the culture supernatant by the measurement of GAG by the DMB assay; **(B) **quantitative analysis of the aggrecan neoepitope CS846 was performed by a specific ELISA assay. The values of the content in cartilage (blue bars) and the cumulative release of GAG/CS846 into the culture medium (red lines) are expressed as means ± SEM. BNC, bacterial nana-cellulose; DMB, dimethylene blue; GAG, glycosaminoglycan; SEM, standard error of the mean; TGF-β1, transforming growth factor-β1.

The same strong degree of Safranin O staining was observed in freshly isolated cartilage and cartilage samples from the entire culture period, indicating negligible loss of proteoglycan (Figure 3 A). There was no obvious difference between non-stimulated and TGF-β1-stimulated samples. Interestingly, initial deposition of negatively charged proteoglycans into BNC adjacent to the cartilage was apparent after eight weeks of culture in TGF-β1-stimulated samples, suggesting a beginning integration of the insert (arrows in Figure 3 A). Quantification of the proteoglycan content in fresh cartilage and cultured cartilage discs using the DMB assay revealed an increased net glycosaminoglycan (GAG) content in non-stimulated cartilage samples compared to fresh cartilage over the entire culture period (Figure 4A). TGF-β1-stimulated cultures showed a higher GAG level than fresh cartilage after two weeks; this decreased during further culture to levels below those of fresh cartilage (Figure 4A). In parallel, cumulative GAG release from cartilage into the supernatant continuously increased throughout *in vitro *culture, indicating a continous, almost linear liberation of proteoglycans over time; this was augmented at all time points by TGF-β1 stimulation (Figure 4A). Interestingly, the cumulative GAG release from cartilage during culture was higher (24.3 mg GAG/g ww non-stimulated; 30.5 mg GAG/g ww TGF-β1-stimulated) than the total content in fresh cartilage tissue (19.2 ± 1.0 mg GAG/g ww), thus illustrating a substantial synthesis capacity of the chondrocytes *in vitro*.

#### Localisation, content, release and transcription of aggrecan

Using an antibody directed against newly synthesized aggrecan molecules, a regenerative response of the cartilage was predominantly detected in chondrocytes at the interface of the cartilage defect and the BNC insert after two weeks of culture (Figure 3B). Interestingly, BNC areas adjacent to the cartilage also exhibited a distinct staining which gradually decreased towards the implant center. In contrast, chondrocytes remote from this area and the interterritorial matrix were not stained.

Upon long-term culture for eight weeks, there was a shift towards a more homogeneous staining of chondrocytes and intercellular matrix throughout the cartilage, approaching the findings in fresh cartilage and, thus, suggesting an attempt to re-establish metabolic tissue homeostasis (Figure 3B). This regenerative response was confirmed by a substantial increase of the CS846 neoepitope content in cartilage samples (approximately two-fold) until two weeks after initiation of culture with a subsequent steady state plateau (Figure 4B). There was no obvious difference between the findings in non-stimulated and TGF-β1-stimulated cartilage. The cumulative CS846 release into the supernatant progressively increased over the entire culture period, with no differences between non-stimulated and TGF-β1-stimulated cartilage samples (Figure 4B). Notably, the total amount of CS846 released from cartilage within eight weeks (approximately 600 μg/g ww) exceeded the total content in fresh cartilage tissue (131 μg/g ww) by a factor of almost five, further underlining the synthesis capacity of the chondrocytes *in vitro*.

**Figure 5:**
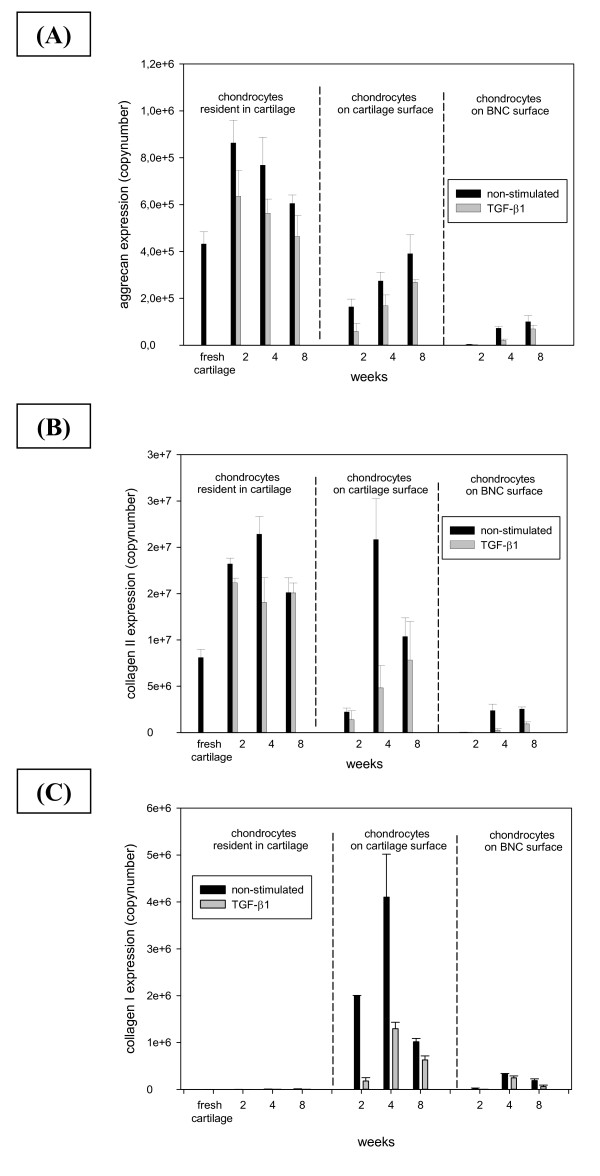
**Real time PCR analysis for Aggrecan, Collagen type II, and Collagen type I-mRNA-expression**. mRNA expression for Aggrecan (**A**), Collagen type II (**B**), and Collagen type I (**C**) was assessed in fresh cartilage, in resident chondrocytes from the matrix of cultured (+/-TGF-β1) cartilage, as well as in cells emigrated from cultured cartilage (+/-TGF-β1) onto the cartilage surface or the BNC matrix. Values are expressed as means ± SEM. BNC, bacterial nana-cellulose; SEM, standard error of the mean; TGF-β1, transforming growth factor-β1.

Differential information on the aggrecan transcription of distinct cell populations located in the cartilage matrix or emigrated either onto the surface of the cartilage or the BNC insert was obtained by real time PCR analysis (Figure 5A). Compared to cells in fresh, non-cultured cartilage, chondrocytes localized in the cartilage matrix displayed an increased aggrecan mRNA expression throughout culture, with a maximum after two weeks and a subsequent decrease over time (Figure 5A). This effect was slightly more pronounced in non-stimulated as compared to TGF-β1-stimulated samples. In contrast, the aggrecan mRNA expression of cells emigrated onto the cartilage surface at two weeks of culture was substantially lower than that in fresh cartilage (Figure 5A) but almost doubled until the eight-week time point, approaching the levels of fresh cartilage. A similar time course was observed in chondrocytes emigrated onto the BNC material; however, the final levels at eight weeks only reached approximately one quarter of those in fresh cartilage (Figure 5A). In general, these effects were more pronounced in non-stimulated than in TGF-β1-stimulated samples.

**Figure 6:**
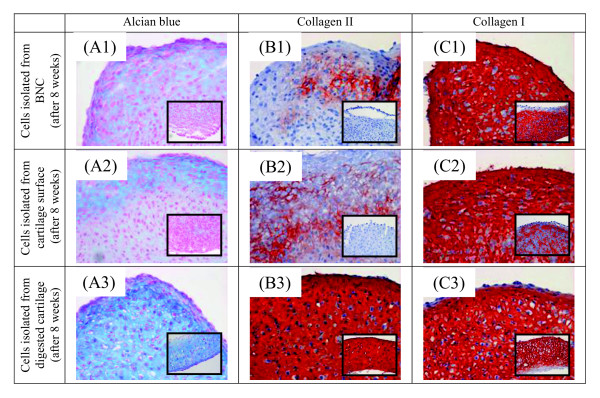
**Histological/immunohistolpogical characterization of high-density pellet cultures of chondrocytes originating from BNC, cartilage surface or enzymatically digested cartilage**. Samples were obtained from cartilage/BNC constructs cultured for eight weeks with continuous TGF-β1 stimulation (identical results for non-stimulated samples). Chondrocytes were then propagated by 'outgrowth-cultures' from isolated BNC inserts or isolated cartilage cylinders, or after enzymatic digestion of isolated cartilage cylinders. After reaching the required amount of cells for the three preparations, high-density cultures of chondrocytes were generated by pellet centrifugation. High-density pellet cultures of chondrocytes originating from BNC **(A1, B1, C1)**, cartilage surface **(A2, B2, C2) **or enzymatically digested cartilage **(A3, B3, C3) **were then subjected to two weeks of culture in chondrogenic (with TGF-β1) or basal medium (without TGF-β1). Sections of high-density pellets were then stained for the appearance of proteoglycans using alcian blue **(A1-A3)**; in addition, collagen type II **(B1-B3) **and type I **(C1-C3) **were identified immunohistologically. Inserts represent the histology of pellets cultured in basal medium. Magnifications: 40 x. BNC, bacterial nana-cellulose; TGF-β1, transforming growth factor-β1.

The increased differentiation of cells on the surface of cartilage discs and BNC inserts towards a chondroid phenotype (as indicated by augmented synthesis of aggrecan mRNA upon long-term culture) was further supported by a substantial deposition of proteoglycan in high-density pellet cultures, approaching the levels observed in the respective cultures of chondrocytes isolated from the cartilage discs (Figure 6A1-A3).

#### Localisation, content, release, translation and transcription of collagen type II

**Figure 7:**
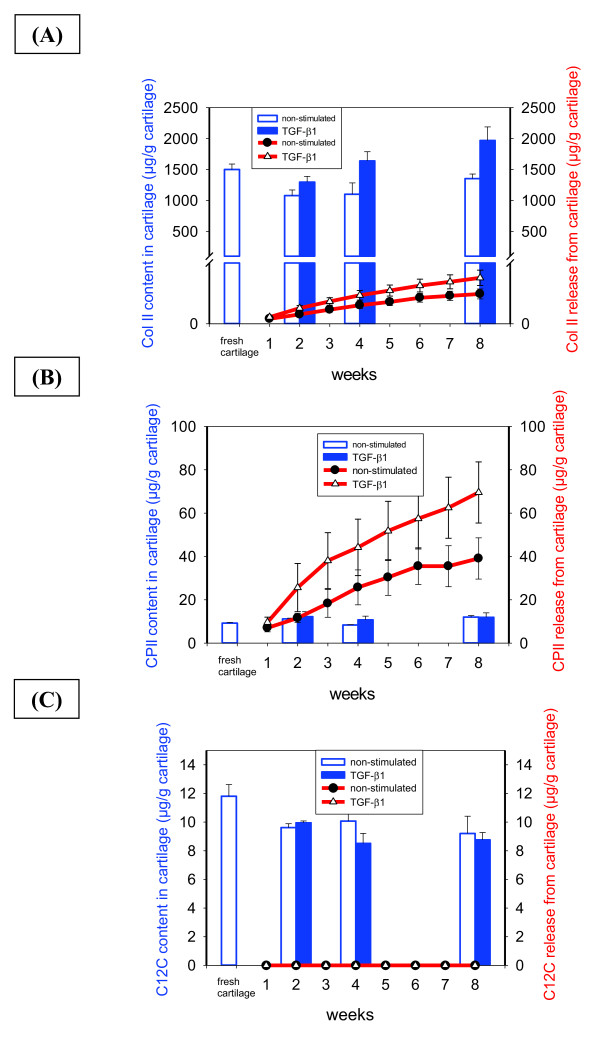
**Quantitative analysis of collagen type II, the collagen type II neoepitope CPII, and the collagen type II degradation-marker C12C**. Collagen type II **(A)**, the collagen type II neoepitope CPII **(B) **and the collagen type II degradation-marker C12C **(C) **were quantitatively assessed in fresh and cultured (+/-TGF-β1) cartilage, as well as in the culture supernatant by specific ELISA assays The values of the content in cartilage (blue bars) and the cumulative release of the respective collagen marker into the culture medium (red lines) are expressed as means ± SEM. BNC, bacterial nana-cellulose; SEM, standard error of the mean; TGF-β1, transforming growth factor-β1.

In both non-stimulated and TGF-β1-stimulated samples and throughout the entire culture period, the cartilage extracellular matrix showed a strong and homogeneous staining for collagen type II, comparable to the staining observed in fresh cartilage (Figure 3C). Clear deposition of collagen type II into the BNC scaffold was observed from two weeks onwards, with steady levels for eight weeks and without any influence of TGF-β1 stimulation. Concordantly, quantitative analysis of the collagen type II content in non-stimulated and TGF-β1-stimulated cartilage discs revealed levels slightly below those of fresh cartilage after two weeks (1,080 and 1,300 μg/g ww versus 1,500 μg/g ww, respectively) and a return to this level at eight weeks (Figure 7A). In contrast to the findings for aggrecan, there was only negligible cumulative release of collagen type II (maximally 3% of the total content in fresh cartilage) from the cultured cartilage discs into the supernatant throughout *in vitro *culture, with higher values in the case of TGF-β1-stimulated cultures versus non-stimulated ones (53 versus 34 μg/g ww; Figure 7A).

As in the case of aggrecan, increased differentiation of cells on the surface of cartilage discs and BNC inserts towards a chondroid phenotype was further supported by initial deposition of collagen type II in high density pellet cultures; however, these levels were clearly below those of the respective cultures of chondrocytes isolated from the corresponding cartilage discs (Figure 6B1-B3).

In agreement with the above findings for collagen type II, an almost steady state level of the precursor molecule procollagen type II was detected in the cartilage discs during the whole culture period, without clear differences in comparison to fresh cartilage or between the findings in non-stimulated and TGF-β1-stimulated cartilage (Figure 7B). The cumulative release of procollagen type II into the supernatant progressively increased over the entire culture period; this was enhanced in TGF-β1-stimulated samples (Figure 7B). In an even stronger fashion than for the aggrecan neoepitope CS846, the total amount of precollagen type II released from cartilage within eight weeks (69 and 31 μg CPII/g ww for TGF-β1- or non-stimulated cartilage discs, respectively) exceeded the total content in fresh cartilage (9.2 ± 0.3 μg/g ww) by a factor of 3.5 to 7.5, on one hand demonstrating a substantial release of the precursor molecule from the cartilage discs, but on the other hand underlining the synthesis capacity of the tissue *in vitro*. In agreement with the relatively stable levels of collagen type II and precollagen II throughout *in vitro *culture, only limited levels of the collagen breakdown marker C12C were detected in either non-stimulated or TGF-β1-stimulated cartilage discs; interestingly, these levels were even lower than those in fresh cartilage (Figure 7C). In addition, the breakdown marker C12C was not detected in the supernatant of any of the *in vitro *cultures (Figure 7C).

As in the case of aggrecan, chondrocytes localized in the cartilage matrix displayed a higher collagen type II mRNA expression than fresh, non-cultured cartilage during the entire culture period, with a maximum after two (TGF-β1-stimulated) or four weeks (non-stimulated) and a subsequent decrease over time (Figure 5B). In contrast, the collagen type II mRNA expression of cells emigrated onto the cartilage surface at two weeks of culture was substantially lower than that in fresh cartilage (Figure 5B), but approached or exceeded the levels in fresh cartilage either at the four-week or eight-week time point. A similar time course was observed in chondrocytes emigrated onto the BNC material; however, as for aggrecan, the final levels of collagen type II mRNA at eight weeks only reached maximally one quarter of those in fresh cartilage (Figure 5B).

In general, these effects were more pronounced in non-stimulated than in TGF-β1-stimulated samples.

### Localisation and transcription of collagen type I

As expected, neither fresh cartilage nor any of the cultured cartilage discs showed a positive staining for collagen type I (Figure 3D). In contrast, staining for collagen I in the BNC inserts progressively increased upon culture, reaching a maximum at eight weeks (Figure 3D). At four and eight weeks, this effect was more pronounced in the non-stimulated cartilage discs.

The mRNA for collagen type I displayed a pattern similar to that observed in immunohistology, that is, the resident cells in fresh or cultured cartilage expressed hardly any collagen type I mRNA, whereas the cells emigrated onto the cartilage surface showed substantial levels of collagen type I mRNA, with peak levels at four weeks (Figure 5C). The induction of mRNA transcription was more pronounced in non-stimulated samples, suggesting an inhibiting effect of TGF-β1. Interestingly, cells emigrated onto the BNC insert showed much lower levels of collagen type I mRNA than those on the cartilage surface, possibly indicating a stabilization of the chondrocyte phenotype upon contact with the BNC (Figure 5C). As for the cells on the cartilage surface, the induction of mRNA transcription was more pronounced in non-stimulated BNC samples.

Strikingly, there were no obvious differences concerning the deposition of collagen type I protein in high-density pellet cultures of cells isolated from the cartilage discs or from the surface of the cartilage or the BNC inserts, indicating a similar degree of dedifferentiation of the individual cell populations in culture (Figure 6C1-C3).

## Discussion

### Suitability of the new model

In the present *in vitro *model for the regeneration of cartilage defects, mature, adult bovine cartilage turned out to be a well-suited tissue source and showed a number of advantages: 1) it is regularly available and allows harvesting of up to 48 cartilage discs per joint with standardized, highly homogenous quality; and 2) the resulting discs show an intact cartilage matrix/surface without structural alterations and/or primary loss of proteoglycans or other matrix molecules, features difficult to achieve with human samples from osteoarthritis or rheumatoid arthritis patients. The resident cartilage cells showed vital morphology for up to eight weeks without any signs of alterations, suggesting that the culture conditions are well-suited to preserve the structural and functional integrity of the chondrocytes. In addition, the matrix integrity of the cartilage seemed to be largely unaffected during culture, except for the well-known detachment of the superficial lamina splendens. This was supported by the long-term, sustained presence of proteoglycans and collagen II. Finally, there were no signs of cartilage dedifferentiation, as underlined by the absence of collagen type I in the cartilage matrix. Thus, the present model seems to provide optimal basic conditions to study the regeneration of injured cartilage in general and appears to be well-adapted for testing the biocompatibility, cell seeding and matrix deposition/regeneration capacity of candidate biomaterials, as shown for the innovative cartilage replacement material BNC. These issues can be monitored by various read-out parameters concerning both the 'host' cartilage and the embedded insert, ranging from the time course of molecule release into the supernatant, structural histological analyses and RNA production, as well as the neo-synthesis, status quo and/or degradation of matrix molecules. Concerning the effects of bioactive factors, TGF-β1 stimulation showed a protective effect on the cartilage matrix integrity, as demonstrated by decreased superficial delamination and emigration of chondrocytes, whereas there was little effect on the BNC insert.

Innovations of the present model in comparison to previous models based on samples derived from immature calves [42,43] or pigs [44] include: 1) the use of adult cartilage, likely more comparable to human diseases with typical adult onset, instead of immature material with a higher regenerative potential; 2) the application of cartilage samples with their physiological surface; and 3) the basic suitability for high-throughput analyses in 48-/96-well plates. On the other hand, limitations of the present model are: 1) the use of bovine instead of human material, with possibile differences in terms of cell density, tissue architecture, and biomechanical properties ([49] and references therein); 2) the application of one-phase 'pure' cartilage constructs instead of two-phase osteochondral contructs, which may be physiologically more meaningful, but may approach the limits of cultivation due to a higher metabolism of the living bone (marrow) component [49-51]; 3) the limited time span in which viable constructs can be maintained in culture without using more complex bioreactors (maximally 12 weeks; data not shown; [44]); 4) the lack of dynamic biomechanical loading of the constructs during culture [51,52]; and 5) the lack of biomechnical testing of the regenerated tissue with push-out or compression tests [42-44,53].

### Integrity of matrix and chondrocytic phenotype in the 'host' cartilage cylinders

The presence of proteoglycans and collagen type II in the cultured 'host' cartilage cylinders remained sustained both at the mRNA and protein level, suggesting optimized culture conditions for the structural and functional integrity of cartilage and chondrocytes. Strikingly, the content of proteoglycan/aggrecan and collagen type II remained comparable to that of fresh cartilage, further underlining the stability of the present *in vitro *system. Proteoglycan and collagen type II levels were maintained despite substantial release of both their mature molecules and neoepitopes into the supernatant, indicating considerable matrix synthesis in the injured cartilage cylinder, as also observed as a repair attempt *in vivo *in osteoarthritis cartilage [54,55]. In the case of collagen type II, the stability of the present model was confirmed by decreased levels of the collagen degradation product C12C, again similarly to the *in vivo *situation of osteoarthritis cartilage [54-56] and similarly to other *in vitro *models [47]. The substantial release of proteoglycan/aggrecan and collagen may be favored by an influence of the *in vitro *conditions on the molecular structure and the resulting binding of the proteoglycans to the cartilage matrix, despite the fact that the high proteoglycan content in the cultured cartilage should protect resident or newly synthesized collagen type II against endogenous proteolytic enzymes [57]. The functional integrity of the cultured cartilage was further underlined by the phenotypic stability of the chondrocyte, that is, the absence of fibroblastic dedifferentiation, such as the expression of collagen type I [58-60].

### Mobilization of chondrocytes from cartilage matrix

Increased delamination in non-stimulated samples was accompanied by augmented migration of cells onto the surface of the cartilage and the BNC implant, suggesting that matrix erosion led to a loosened network around the chondrocytes and active emigration of the cells. This is most likely an *in vitro *'artifact' upon extended culture of the cartilage and the emigration appears to occur predominantly out of and onto the surface of the cartilage cylinders. The general migration capacity of chondrocytes has been previously described in isolated cells [61-64]. In the case of osteoarthritis or traumatized cartilage, a focused loss of proteoglycans and/or collagens is believed to favor the egress of cells from the matrix [65-67]. Thus, both superficial delamination and loss of matrix molecules may have contributed to the emigration of chondrocytes in the present model.

### Matrix formation in the biomaterial BNC

During the first two weeks, newly synthesized aggrecan was predominantly produced in chondrocytes adjacent to the defect with a clear diffusion into the neighboring BNC implant. A primary sealing of a defect area contributing to a reduction of the defect size *in vivo *is known as 'cartilage flow-phenomena' [68,69]. In i*n vitro *models, however, the active synthesis of new matrix occurs independently of biomechanical loading. The concurrent detection of mRNA and protein for cartilage-specific aggrecan and collagen type II (in the case of the protein throughout the BNC insert), underlines the suitability of the present model, the biocompatibility of the BNC, and the high synthetic capacity of the cartilage-resident or emigrated chondrocytes [70,71]. An initial suppression and subsequent partial recovery of the mRNA expression for aggrecan/collagen type II in cells migrated onto the surface of the cartilage or the BNC implant - a phenomenon well-known for chondrocytes expanded in monolayer culture and then transferred to three-dimensional culture [60,72-74] - further supports these assumptions.

### Dedifferentiation/redifferentiation of chondrocytes on the BNC surface

Chondrocytes emigrated onto the BNC surface showed certain signs of dedifferentiation, such as a fibroblastic phenotype, as well as higher expression of collagen type I mRNA and lower mRNA expression for aggrecan/collagen type II mRNA than in fresh cartilage [59,60,72-75]. It has to be taken into account, however, that a transient dedifferentiation may be beneficial for the recruitment of the cells from the cartilage matrix [64]. On the other hand, there were also indications of a successful redifferentiation of the emigrated cells upon contact with the BNC surface. These included an increase of the mRNA for aggrecan/collagen type II over time and substantially decreased levels of collagen type I mRNA compared to those in condrocytes on the cartilage surface [76]. This suggests that BNC, as already observed for other biomaterials [75,77-79], is capable of stabilizing the chondrocytic phenotype. This was further supported by a substantial initial deposition of proteoglycan and collagen type II by the cells on the BNC surface in long-term high-density pellet cultures.

### Relative impact of TGF-β1

Interestingly, TGB-β1 stimulation showed a long-lasting, protective effect on the matrix integrity, as demonstrated by decreased/delayed superficial delamination and emigration of chondrocytes. This may be due to the induction of lubricin, a major component of the cartilage surface-covering lamina splendens [80-82], the suppression of matrix degrading enzymes, such as matrix metalloproteinases (MMP) [83], and concurrent up-regulation of their inhibitors [84,85] and/or induction of matrix synthesis [33,34,36].

Differential effects of TGF-β1 stimulation on other parameters were restricted to an opposing influence on the content of the cartilage matrix markers aggrecan and collagen type II, as previously described [38-40]. The limited influence of TGF-β1 stimulation is probably due to the fact that serum starvation, normally used to enhance the effects of subsequent growth factor stimulation, severely damages the 'host' cartilage cylinder and, therefore, cannot be applied to the present long-term model.

### Bacterial nanocellulose as a potential cartilage implant material

In the present model, the cell-free, non-resorbable cartilage replacement material BNC proved highly suitable in supporting early stages of matrix formation in the cartilage defects. This was underlined by: 1) smooth adaptation of the BNC to the defect edges in the 'host' cartilage cylinder, likely based on the enormous water binding and swelling capacity of BNC and generally considered a prerequisite for successful cartilage regeneration [86,87]; 2) emigration/seeding of the BNC with resident, phenotypically stable chondrocytes without any signs of toxicity, indicating a high biocompatibility of the material; 3) substantial *de novo *deposition of cartilage-specific matrix onto and into the BNC scaffold, contributing to the sealing of the defect; and 4) initial signs of lateral integration/bonding of the BNC (in)to the edges of the cartilage defect, indicated by the so-called 'cartilage flow phenomenon' and also regarded as pivotal for defect regeneration *in vivo*. These findings are in agreement with the known biocompatibility of BNC as a scaffold material in general [11,12,88-90] and, in particular, its capacity to support the growth of vital, metabolically active chondrocytes [20].

Strikingly, all the above-mentioned, favorable features of the biomaterial BNC were achieved with a cell-free preparation, theoretically eliminating the need of cell harvesting with inevitable damage to healthy cartilage *in vivo *and allowing storage as an off-the-shelf product. In addition, the positive results were generated with a non-resorbable biomaterial, allowing the long-term formation of a BNC-cartilage matrix composite *in vivo *and, possibly, limiting adverse reactions due to rapid release of breakdown products [91].

Notably, there was no immigration of chondrocytes in the central area of the BNC, possibly due to the relatively small diameter of the pores in the BNC network (2 to 5 μm), compared to the cell diameter (10 to 20 μm). This problem may be addressed by modified network structures, enabling three-dimensional seeding with chondrocytes [92]. Since there were very little, if any, differential effects of TGF-β1 stimulation on the matrix formation in the BNC (as also observed for the cartilage cylinders), the usefulness of TGF-β1 coating remains to be finally assessed.

## Conclusions

The present long-term *in vitro *model with mature, adult bovine cartilage is highly suitable for the testing of cartilage regeneration with candidate biomaterials, based on: 1) the quasi unlimited availability, reproducible quality and extended tissue integrity of the 'host' bovine cartilage cylinders; 2) successful seeding of the biomaterial (in this case BNC) with phenotypically stable chondrocytes; and 3) substantial *de novo *deposition of cartilage-specific matrix onto and into the biomaterial scaffold. This represents a robust, economic and versatile system to analyze thoroughly the interaction and reciprocal effects of cartilage and biomaterial with a broad spectrum of morphological and molecular techniques.

Using this model, BNC was identified as a promising biomaterial for supporting early stages of matrix formation in cartilage defects. This was achieved with a cell-free BNC preparation, possibly avoiding previous harvesting of chondrocytes and allowing long-term storage as a stable product. Its non-resorbable character may favor the formation of a durable BNC-cartilage matrix composite *in vivo*, without limitations due to a slowly regenerating cartilage matrix.

The necessity to add bioactive factors to the BNC, and in particular the molecular nature of such factors, will be the focus of future studies.

## Abbreviations

BNC: bacterial nanocellulose; BSA: bovine serum albumin; DAB: diaminobenzidine tetrahydrochloride; DMB: dimethylene blue; (D)MEM: (Dulbecco´s) modified Eagles´s medium; EDTA: ethylenediaminetetraacetic acid; ELISA: enzyme-linked immunosorbent assay; GAG: glycosaminoglycan; Hestrin and Schramm; IgG: immunoglobulin G; ITS: insulin-transferrin-selenium; MACT: matrix-assisted autologous chondrocyte transplantation; PBS: phosphate-buffered saline; SEM: scanning electron microscopy; TGF: transforming growth factor.

## Competing interests

The authors declare that they have no competing interests.

## Authors' contributions

Study conception and design: DP, SL, CK, RWK. Acquisition of data: DP, SL, HA, ME. Analysis and interpretation of data: DP, SL, HA, ME, CK, DK, RWK. Drafting and/or critical revision of the manuscript: DP, SL, HA, ME, CK, DK, RWK. All authors read and approved the final manuscript.

## Supplementary Material

Additional file 1 **Scanning electron micrographs of BNC (A) and bovine cartilage (B)**. Note the apparent ultrastructural similarity of the three-dimensional BNC network and the cartilaginous collagen fibers. Magnification: 5,000x. Click here for file

Additional file 2 **HE-stained vertical and cross sections of a cartilage cylinder containing a BNC insert**. Note the tight bonding of the BNC material to the surrounding cartilage edges. Magnification: 40 x. Click here for file
