# Supplementary figures and images for: A novel in vitro bovine cartilage punch model for assessing the regeneration of focal cartilage defects with biocompatible bacterial nanocellulose
Source: Arthritis Res Ther. 2013 May 14;15(3):R59. doi: 10.1186/ar4231 (PMC4060236; doi:10.1186/ar4231)

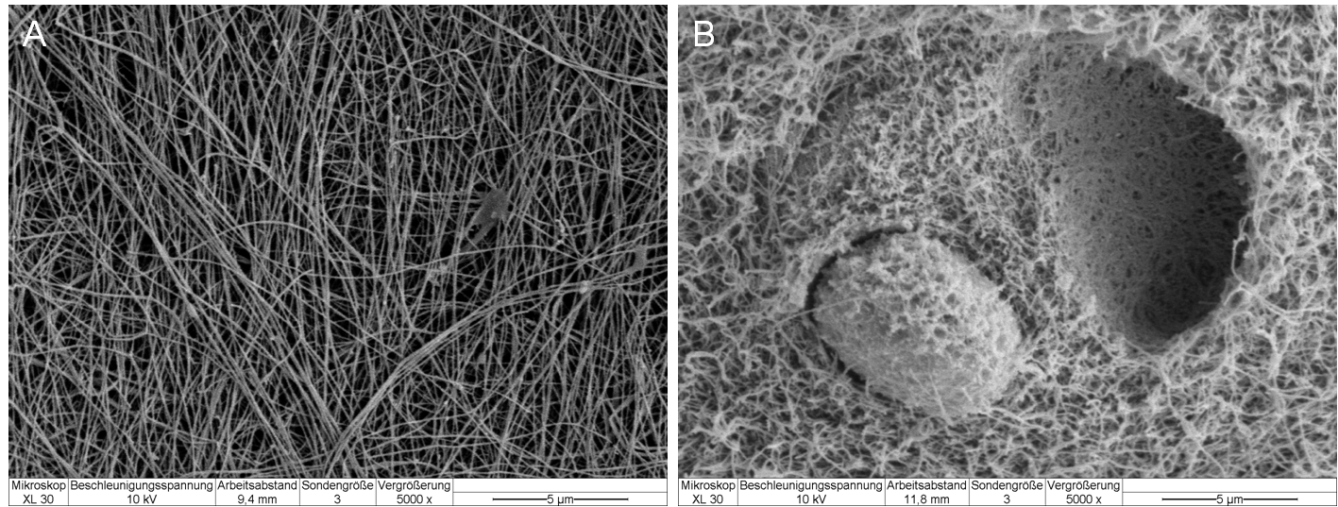


**Additional file 1**

Supplement: Additional file 1 — Scanning electron micrographs of BNC (A) and bovine cartilage (B). Note the apparent ultrastructural similarity of the three-dimensional BNC network and the cartilaginous collagen fibers. Magnification: 5,000x. [file ar4231-S1.DOCX]

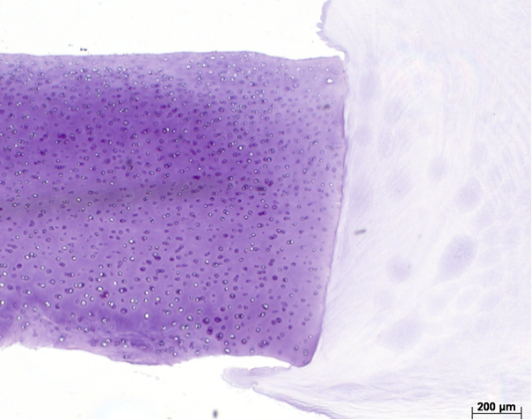

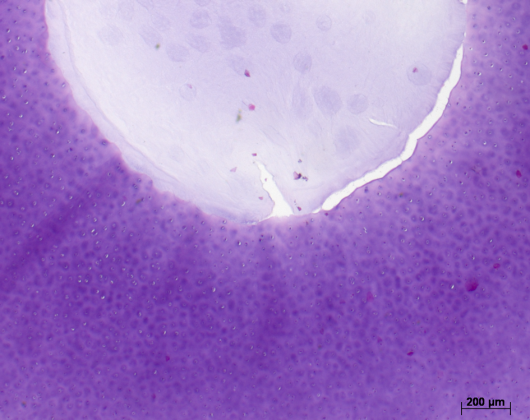


**Additional file 2**

Supplement: Additional file 2 — HE-stained vertical and cross sections of a cartilage cylinder containing a BNC insert. Note the tight bonding of the BNC material to the surrounding cartilage edges. Magnification: 40 x. [file ar4231-S2.DOCX]
